# Multispecies facial detection for individual identification of wildlife: a case study across ursids

**DOI:** 10.1007/s42991-021-00168-5

**Published:** 2022-04-07

**Authors:** Melanie Clapham, Ed Miller, Mary Nguyen, Russell C. Van Horn

**Affiliations:** 1 Department of Geography, University of Victoria, 3800 Finnerty Road, Victoria, BC V8P 5C2 Canada; 2 BearID Project, Sooke, BC Canada; 3 San Diego Zoo Wildlife Alliance, San Diego, CA USA

**Keywords:** Bears, Deep learning, Face recognition, Individual ID, Machine learning, Ursidae

## Abstract

To address biodiversity decline in the era of big data, replicable methods of data processing are needed. Automated methods of individual identification (ID) via computer vision are valuable in conservation research and wildlife management. Rapid and systematic methods of image processing and analysis are fundamental to an ever-growing need for effective conservation research and practice. Bears (ursids) are an interesting test system for examining computer vision techniques for wildlife, as they have variable facial morphology, variable presence of individual markings, and are challenging to research and monitor. We leveraged existing imagery of bears living under human care to develop a multispecies bear face detector, a critical part of individual ID pipelines. We compared its performance across species and on a pre-existing wild brown bear *Ursus arctos* dataset (*BearID*), to examine the robustness of convolutional neural networks trained on animals under human care. Using the multispecies bear face detector and retrained sub-applications of *BearID*, we prototyped an end-to-end individual ID pipeline for the declining Andean bear *Tremarctos ornatus*. Our multispecies face detector had an average precision of 0.91–1.00 across all eight bear species, was transferable to images of wild brown bears (AP = 0.93), and correctly identified individual Andean bears in 86% of test images. These preliminary results indicate that a multispecies-trained network can detect faces of a single species sufficiently to achieve high-performance individual classification, which could speed-up the transferability and application of automated individual ID to a wider range of taxa.

**Supplementary Information:**

The online version contains supplementary material available at 10.1007/s42991-021-00168-5.

## Introduction

Conservation technologies can enhance the collection, analysis and sharing of wildlife-related data, and the implementation and evaluation of global conservation action (Lahoz-Monfort et al. 2019). Computer vision within ecology and conservation increasingly facilitates the description of image features, counting within images, and identity classification (Weinstein 2018). This automated approach enables rapid processing and classification of large datasets in a standardized way that will be vital for global data sharing (Steenweg et al. 2017; Ahumada et al. 2020). Machine learning, and more specifically deep learning techniques, are now a focus of image classification, with emphasis on species and individual identification (ID) (Christin et al. 2019).

Computer vision enhances the integration of individual ID into ecological research, with the benefit of images being sourced from camera trap surveys and citizen science/ecotourists, allowing researchers to monitor species over broader scales (Berger-Wolf et al. 2017; Araujo et al. 2019; Schneider et al. 2019; Nipko et al. 2020). Individual photo ID has primarily focused on species with unique, stable body markings, such as the Grévy’s zebra *Equus grevyi* (Crall et al. 2013) and the Northern giraffe *Giraffa camelopardalis* (Miele et al. 2020), other morphological traits and scars (Kelly and Holub 2008), or body parts such as fin shape (Hughes and Burghardt 2017). Face recognition is an alternative method of visual individual ID, especially when species lack distinguishing markings. Originally built for chimpanzees *Pan troglodytes* based on human facial recognition approaches (Loos and Ernst 2013), it has now been applied to other primates and selected large mammals (Deb et al. 2018; Körschens et al. 2018; Chen et al. 2020; Guo et al. 2020; Clapham et al. 2020), taking advantage of advances in deep learning techniques (Ravoor and T.S.B. 2020). Deep learning networks require large, labelled datasets that are difficult to acquire for wild animals, resulting in networks being trained primarily using images of individuals under human care (e.g., Freytag et al. 2016; Chen et al. 2020). Currently, a tradeoff exists between the need for reliable training data of individuals and the need for robust training data that reflects the contexts in which deep learning networks will be used (see Beery et al. 2018); the former favouring images taken ex situ and the latter favouring images of wild animals in situ (Schofield et al. 2019; Clapham et al. 2020). A possible solution is to train networks that generalise across species, sourcing larger datasets with diverse facial characteristics and background context, which may increase the robustness of ex situ-trained networks.

Bears (ursids) present an important focal taxonomic family for examining the application of computer vision to wildlife ecology, as they represent lineages that have diverged ecologically and morphologically over ~ 12.5 million years (Kutschera et al. 2014), inhabit habitats ranging from ice floes and deserts to forests, they are elusive, generally solitary and do not defend strict territories (Penteriani and Melletti 2020), which makes them challenging to research and monitor compared to other large carnivores. In addition, bear conservation and management would benefit from improved research tools. Six of the eight extant species of bear are considered vulnerable to extinction, with decreasing population trends for four of these six species (Scotson et al. 2017; Velez-Liendo and García-Rangel 2017; Dharaiya et al. 2020; Garshelis and Steinmetz 2020). Knowledge gaps exist across species, particularly for declining species across Asia (Asiatic black bear *Ursus thibetanus,* sun bear *Helarctos malayanus*, sloth bear *Melursus ursinus*) and South America (Andean bear *Tremarctos ornatus*). Tools in machine learning, coupled with monitoring techniques such as camera trapping, could help researchers to generate knowledge on population size, demography, and behaviour (Christin et al. 2019).

Within the *Ursidae*, the presence of distinguishing marks varies by species; Andean bears, Asiatic black bears, sun bears, and to some extent giant pandas *Ailuropoda melanoleuca*, generally possess varied markings on the face or chest that human observers have used for visual identification of individuals (Higashide et al. 2012; Ngoprasert et al. 2012; Zheng et al. 2016; Molina et al. 2017; Appleton et al. 2018; Penteriani et al. 2020; Rodríguez et al. 2020; Morrell et al. 2021), whereas American black bears *U. americanus*, brown bears *U. arctos*, polar bears *U. maritimus*, and sloth bears generally do not (although see Shimozuru et al. 2017). However, there is concern regarding the standardization of manual individual ID approaches across different researchers, for wildlife with and without distinguishing markings (Choo et al. 2020; Johansson et al. 2020). In addition, markings may not be entirely stable over time for some species (Yoshizaki et al. 2009; Van Horn et al. 2015) or easily distinguished under different environmental conditions from photographs (Reyes et al. 2017), and visual individual ID is labor-intensive, especially as multiple trained observers may be required (Ramsey et al. 2019; Morrell et al. 2021). Automated individual ID was recently developed for brown bears (Clapham et al. 2020) and giant pandas (Chen et al. 2020) using deep-learning approaches of facial recognition. Deep convolutional neural networks (CNN) could make use of distinguishing facial markings depending on their stability, but also benefit from other biometric features of an animal’s face. Automated facial recognition may provide a standardized and reproducible approach of individual ID across multiple taxa that could be more broadly accessible to researchers.

We used deep-learning techniques to develop a multispecies facial detector across all eight members of the *Ursidae* family. We used this multispecies detector, trained on images of bears at zoos and rescue centers, to: (1) evaluate the variation in its performance for each ursid species, (2) compare its performance to a single-species trained detector on a pre-existing test set of wild brown bears, and (3) develop an example end-to-end pipeline in combination with a retrained face encoder and SVM (classifier) for automated individual ID of a novel species, the Andean bear. We compared results to those presented in Clapham et al. (2020), where networks were trained and tested on wild brown bears. A multispecies facial detector could reduce development time and advance the application of automated individual ID across a broader range of species. In practice, face detectors could be linked to pre-existing animal object detectors and species classifiers, to pull images of focal species for individual ID.

## Methods

### Data collection

**Fig. 1:**
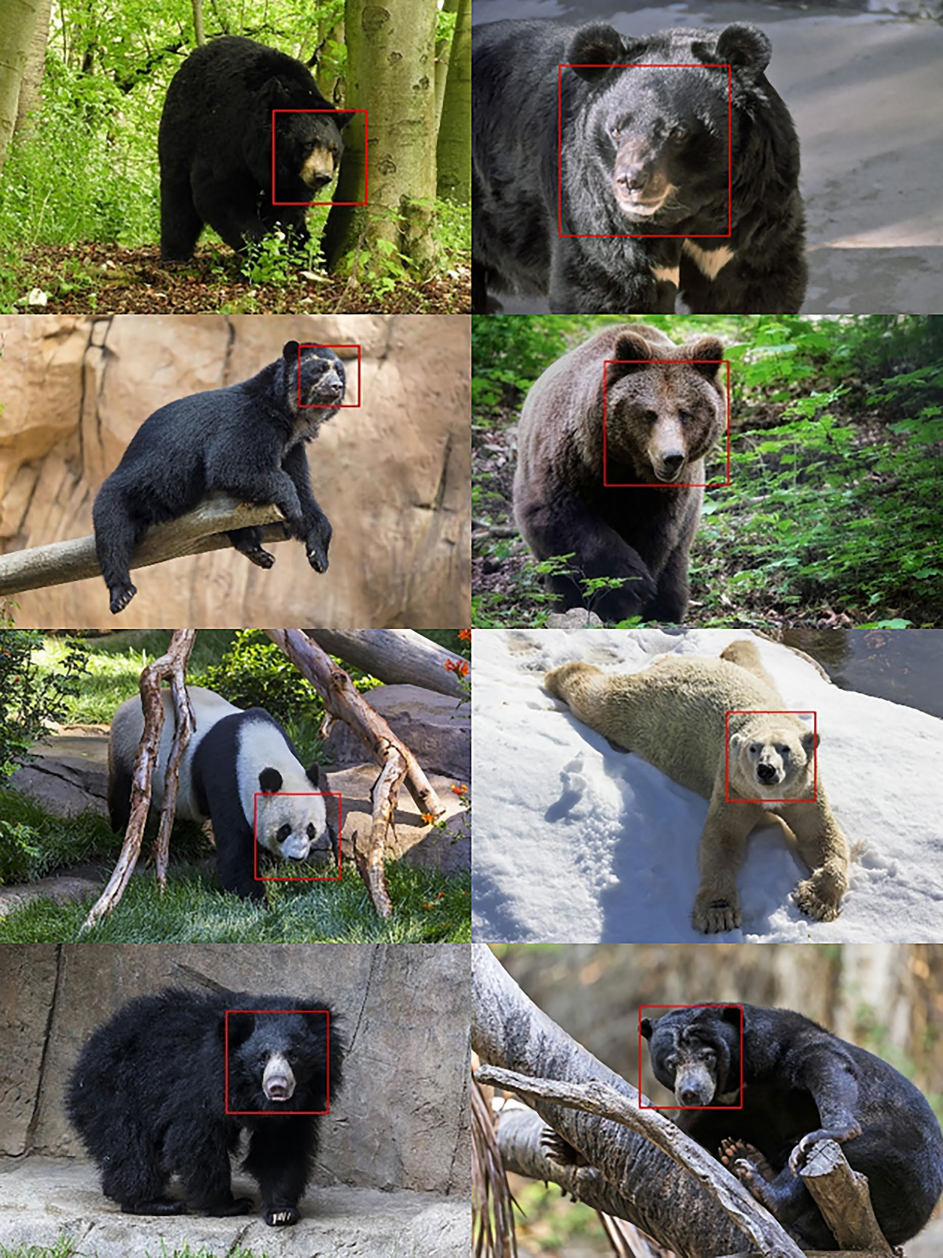
Example images showing variation of captive bears images across all eight species. Red boxes indicate faces detected using the multispecies face detector. Species from top left to bottom right: American black bear, Asiatic black bear, Andean bear, brown bear, giant panda, polar bear, sloth bear, and sun bear

Images were sourced from bears housed in zoos and sanctuaries across North America, Europe, and Asia. Images were initially collected for separate research (Van Horn et al. 2014, 2015), and the dataset was supplemented for this study (Table 1). The only criteria for image inclusion was that both eyes of the bear needed to be visible for the object detector to find the face (see below) of a bear of known identity. Identification of individual bears were provided by the image source, with bear birthdate and sex sometimes sourced from individual zoos or, most commonly, from studbooks. The habitat of bear enclosures varied across the dataset, creating variation in the background of images (Fig. 1). The dates on which images were taken were sourced from the image metadata if present. The exact cameras (brand and model) used to take the photographs were unknown due to the post hoc nature of image collection, however, image resolution ranged from 0.06 to 3.84 megapixels. Images were JPEG, PNG and TIFF format. TIFF images were converted to JPEG prior to use.

**Table 1:** Summary of multispecies image dataset

Species	Number of face images (face chips)	Number of individuals	Mean face chips per individual
American black bear *Ursus americanus*	84	22	3.8
Andean bear *Tremarctos ornatus*	609	32	19.0
Asiatic black bear *U. thibetanus*	54	25	2.2
Brown bear *U. arctos*	588	49	12.0
Giant panda *Ailuropoda melanoleuca*	185	73	2.5
Polar bear *U. maritimus*	481	175	2.8
Sloth bear *Melursus ursinus*	92	34	2.7
Sun bear *Helarctos malayanus*	99	35	2.8
Total	2192	445	4.9

#### Andean bear subset

We used the Andean bear subset (Table 1) for inclusion in our end-to-end example application. To minimize the impact of ontogenetic changes in morphology on overall success, only images of Andean bears over 2 years of age were included in the dataset, and only individuals with seven or more images were included to allow sufficient number of images for testing and training. This allowed a minimum number of two images per individual available for testing, a requirement of the testing methodology for evaluating similarity comparison networks (see “Testing methodology”). Images of Andean bears were taken from 2004 to 2021, with a mean span of 7.4 (± SD 4.1) years per individual. Eight percent of total images were missing information for the year the image was taken. Of the individual bears included in the dataset, 13 were female and 19 were male. We used a randomly selected 80/20% split of the 609 images for training (*n* = 488) and testing (*n* = 121), respectively.

### Golden dataset

The golden dataset is the manually annotated ‘gold standard’ dataset (*n* = 2192) and is used for training networks (with the training dataset) and evaluating their performance (with the testing dataset). We ran all images through the application *bearface* (Clapham et al. 2020) to speed-up creating a golden dataset, and manually adjusted erroneous or missing bounding boxes and facial landmarks using *imglab* from the Dlib toolkit (King 2009). The golden dataset also included labels of individual identification for the Andean bear subset.

### Multispecies face detector development

We trained a multispecies face detector network (*face_allbears.dat*), using the sub-application (*bearface*) from *BearID* developed by Clapham et al. (2020), to evaluate its performance across species and on an existing wild brown bear dataset. We split the multispecies dataset (Table 1) into 80% for training and 20% for testing within species, and then combined these together across species, resulting in 1754 images for training and 438 for testing. The *BearID* pipeline is based on the FaceNet approach (Schroff et al. 2015) and consists of: (1) face detection (*bearface* sub-application), (2) face reorientation and cropping (*bearchip* sub-application), (3) face encoding (*bearembed* sub-application) and (4) face classification (*bearsvm* sub-application) (see Clapham et al. 2020 for a full description of the *BearID* application pipeline and hardware used). For this study, we used Microsoft Azure NC6s_v3 cloud computing instances to train and evaluate the multispecies face detector.

*Bearface* with the new network *face_allbears.dat*, hereafter *bearface*, finds faces and facial landmarks (i.e., the outer corners of the eyes) of multiple bear species in images. As in Clapham et al. (2020), it consists of an object detector (sliding window and CNN (Dalal and Triggs 2005; King 2015)) and a shape predictor [face alignment with an ensemble of regression trees (King 2009; Kazemi and Sullivan 2014)]. The object detector and shape predictor were trained using the bounding boxes and facial landmark labels from the golden dataset. See Clapham et al. (2020) for full equivalent training procedures. *Bearface* accepts JPEG and PNG file types as input images (other formats would need pre-conversion), and outputs an XML file that includes a list of the images with predicted face and landmark data for each.

### End-to-end Andean bear example pipeline

**Fig. 2:**
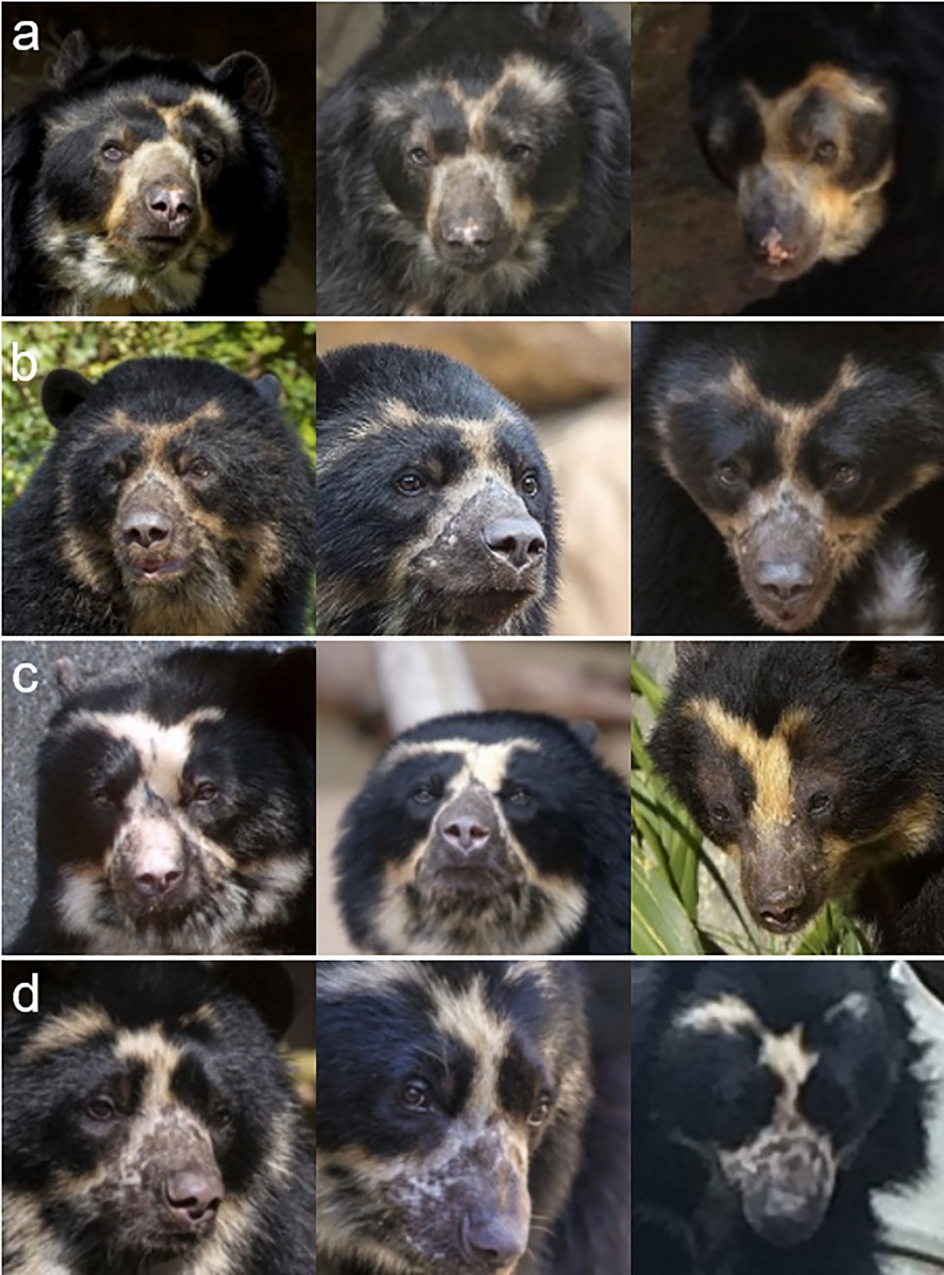
Example face chips produced by the multispecies detector (*bearface*) and the reorientation/cropping application (*bearchip*); **a**–**d** different individual Andean bears displaying variation in facial appearance within and among individuals, **a** and **b** are males, while **c** and **d** are females

**Fig. 3:**
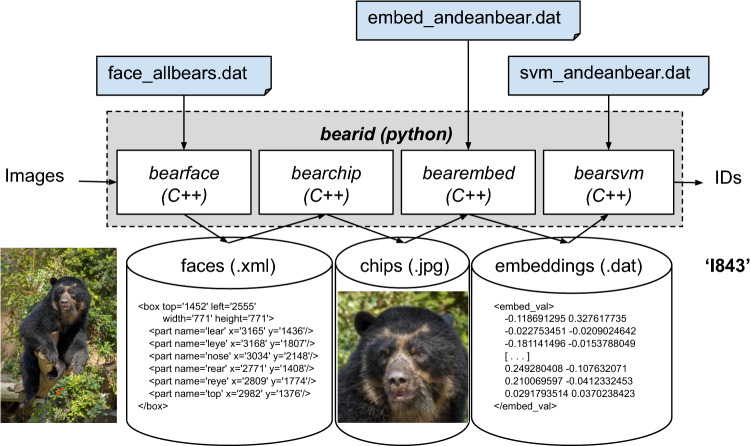
Schematic of *BearID* pipeline including programming languages and file formats, adapted from Clapham et al. (2020). Blue boxes indicate the new networks trained in this study. The process begins with an input image, the face of the bear is detected using the multispecies network of *bearface*, the face is cropped and rotated using *bearchip*, an embedding is created using the Andean bear network of *bearembed*, the embedding is then matched using Andean bear *bearsvm* to produce a classification and output individual ID, e.g., Andean bear ‘I843’)

We used the Andean bear subset of the golden dataset with the multispecies face detector and the pre-existing bear recognition pipeline (*BearID*: Clapham et al. 2020), maintaining the same 80/20% split of the data for training and testing, respectively. *Andean BearID* (*BearID* with new networks), consists of four sub-applications: (1) *Bearface* finds bear faces, (2) *Bearchip* creates face chips from found faces (Fig. 2), (3) *Bearembed* creates embeddings for the face chips, and (4) *Bearsvm* determines individual ID from embeddings (Fig. 3). *Bearface* uses *face_allbears.dat* network, which required no additional training from that described in the previous section. Both the face encoder (*bearembed*) and face classifier (*bearsvm*) required individual-specific retraining with Andean bear images. *Bearchip* required no retraining. Face chips produced by *bearchip* (Fig. 2) using the Andean bear subset of the golden dataset were used to retrain the similarity comparison network *bearembed*, resulting in a new face embedding network, *embed_andeanbear.dat* (Fig. 3). The embeddings produced by that network were used to retrain *bearsvm*, resulting in a new face classifier network, svm_andeanbear.dat (see Code Availability for all code required for retraining networks). Hereafter, *bearembed* and *bearsvm* refer to their use collectively with the Andean bear-trained networks.

### Testing methodology

#### Multispecies face detector

We followed the testing methodology of Clapham et al. (2020) and tested the object detector and shape predictor separately. We focus on interpolated average precision (area under a precision-recall curve) as a focal performance metric for the face detector overall, but also present precision $\left(\frac{\mathrm{true positive}}{\mathrm{true positive }+\mathrm{false positive}}\right)$ and recall $\left(\frac{\mathrm{true positive}}{\mathrm{true positive }+\mathrm{false negative}}\right)$ . We tested performance of the facial detector across the trained species (Table 1), as well as on the test set of wild brown bears from Clapham et al. (2020) (*n* = 934), to examine the performance of a captive-trained detector on wild-tested images.

#### Andean bear end-to-end pipeline

We tested the full end-to-end Andean bear application from input file to ID classification, but also considered the performance of sub-applications *bearembed* and *bearsvm* separately, to assess performance without cumulative error by comparing results to labels from the golden dataset. The Andean bear face encoder (*bearembed*) was tested using pairs of images generated from the test split of the golden dataset (*n* = 121). The paired test set represented 230 matching pairs of images (same individual) and 230 non-matching pairs (different individual). There were even numbers of matching and non-matching pairs, no face chip was compared to itself, and each pair within the test set was unique. We further evaluated the predictive capability of the embedding network for both known individuals and unknown (or new) individuals by applying fivefold validation across two test regimes:

1. Folds across all face chips, whereby different chips for the same individual appear in every fold. Paired tests represent 214 matching and 214 non-matching pairs.
2. Folds by ID label, whereby all chips from an individual appear in only one fold. Paired tests represent 400 matching and 400 non-matching pairs.

We evaluated performance using accuracy: $\left(\mathrm{true\,positive\,rate}\times \mathrm{positive\,ratio}\right)+\left(\mathrm{true\,negative\,rate} \times \mathrm{negative\,ratio}\right)$, positive and negative ratio = 0.5, and F1 score: $2\times \left(\frac{\mathrm{precision }\times \mathrm{ recall}}{\mathrm{precision }+\mathrm{ recall}}\right)$, which is the harmonic mean of precision and recall. Precision here refers to correctly identified matching pairs from all predicted matching pairs. Recall refers to correctly identified matching pairs out of all the actual matching pairs. F1 score may be a preferred metric of performance when classes are imbalanced. In all cases, we used a closed-set approach (Deb et al. 2018). *Bearsvm* was evaluated by comparing the test set accuracy (number of correct ID predictions/total number of ID predictions) of predicted ID labels to those in the golden dataset.

## Results

### Multispecies face detector

The average precision (intersection over union = 0.5) of the multispecies object detector varied among bear species, with an overall performance across species of 0.959 (Table 2). The overall mean normalised distance between the facial landmarks of the golden dataset and those predicted by the shape predictor was 0.083 ± 0.115 (Table 2); in other words, ~ 8% of the distance between the outer corners of the eyes.

**Table 2:** Testing performance of the multispecies-trained facial detector

Test species	Test *n*	Precision (OD)	Recall (OD)	Average precision, IoU = 0.5 (OD)	Mean (± SD) normalised distance (SP)
American black bear	17	1.000	1.000	1.000	0.144 ± 0.188
Andean bear	121	0.975	0.983	0.978	0.061 ± 0.057
Asiatic black bear	11	1.000	0.909	0.909	0.150 ± 0.148
Brown bear	118	0.983	0.966	0.966	0.085 ± 0.103
Giant panda	37	1.000	0.973	0.973	0.103 ± 0.229
Polar bear	96	0.989	0.979	0.969	0.072 ± 0.090
Sloth bear	18	0.947	1.000	1.000	0.127 ± 0.122
Sun bear	20	0.905	0.950	0.920	0.091 ± 0.080
All species	438	0.975	0.970	0.959	0.083 ± 0.115

*OD *object detector, *SP *shape predictor, *IoU *intersection over union

**Fig. 4:**
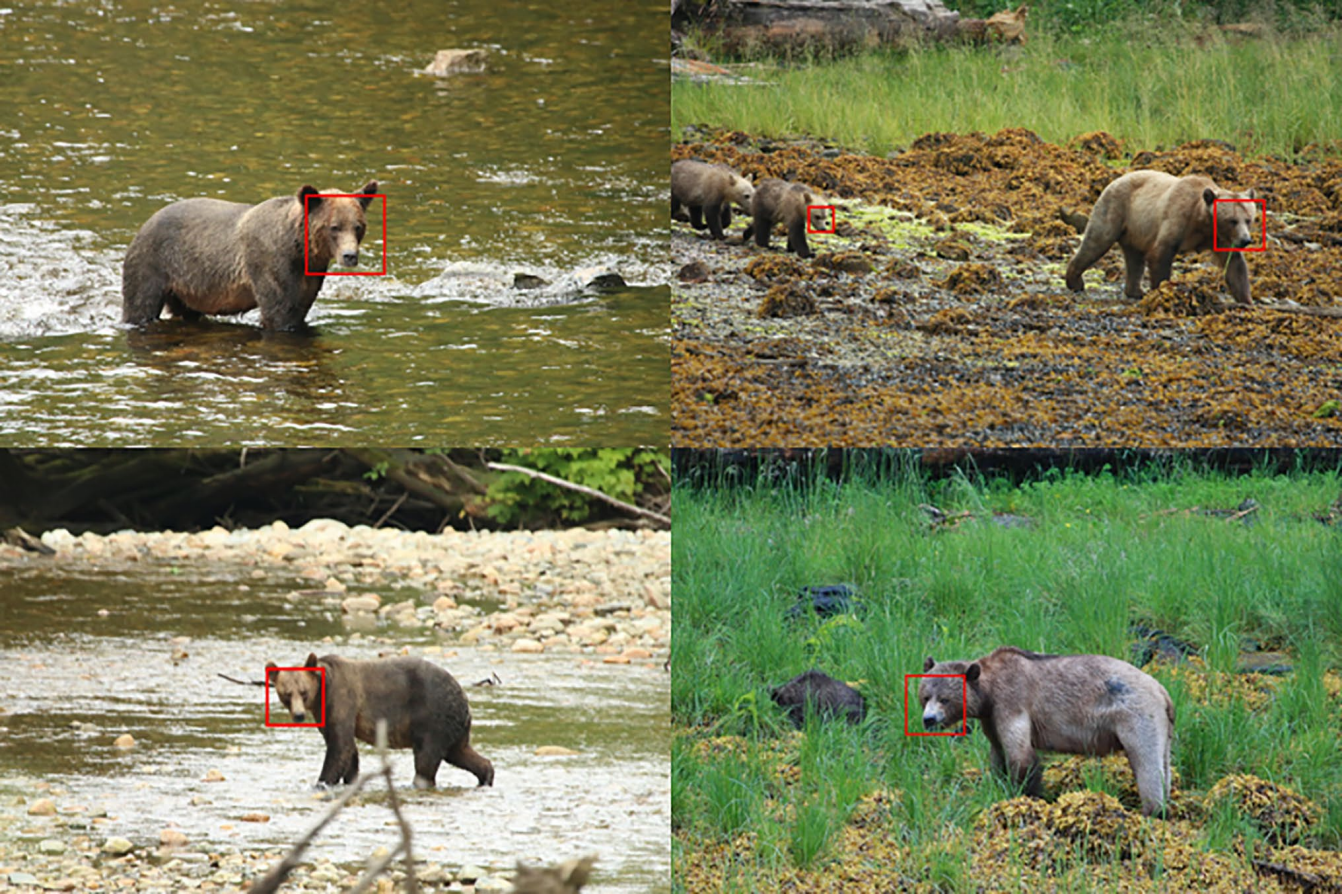
Automated face detection (red boxes) using the multispecies face detector on images of wild brown bears

The average precision of the multispecies object detector (trained on captive bears) when tested on a wild brown bear dataset was 0.929, which is similar to the performance of a detector trained on a wild brown bear dataset (Table 3; Fig. 4). For the wild brown bear dataset tested with the multispecies detector, the mean normalised distance between the facial landmarks of the golden dataset and those predicted by the shape predictor was 0.161 ± 0.155 (Table 3); ~ 16% of the distance between the outer corners of the eyes.

**Table 3:** Comparing the testing performance of the multispecies detector (trained on images of captive bears) on images of wild brown bears and to results from a species-specific detector

Trained detector	Test set	Test *n*	Precision (OD)	Recall (OD)	Average precision, IoU = 0.5 (OD)	Mean (± SD) normalised distance (SP)
Multispecies (captive-trained)	Wild brown bear^a^	934	0.980	0.939	0.929	0.161 ± 0.155
Brown bear (wild-trained)^b^	Wild brown bear	934	0.986	0.983	0.977	0.111 ± 0.122

*OD *object detector, *SP *shape predictor, *IoU *intersection over union

^a^Represents a wild bear dataset (from Clapham et al. 2020) tested on a captive bear-trained detector

^b^Baseline comparative results from Clapham et al. (2020)

### End-to-end Andean bear pipeline

Using the multispecies detector network with *bearface* (Table 3), the original *bearchip* and the newly trained networks for *bearembed* and *bearsvm*, the end-to-end Andean bear pipeline correctly predicted the ID for 104 out of 121 images (closed-set accuracy = 86.0%). *Bearface* detected 120 of 121 Andean bear faces, plus 2 erroneous faces were detected in one image for a total of 122 faces detected. We manually removed these erroneous detections from the pipeline at this stage to maintain an accurate evaluation of *bearembed* and *bearsvm*. Of the 120 correctly detected faces, 104 were correctly identified.

**Fig. 5:**
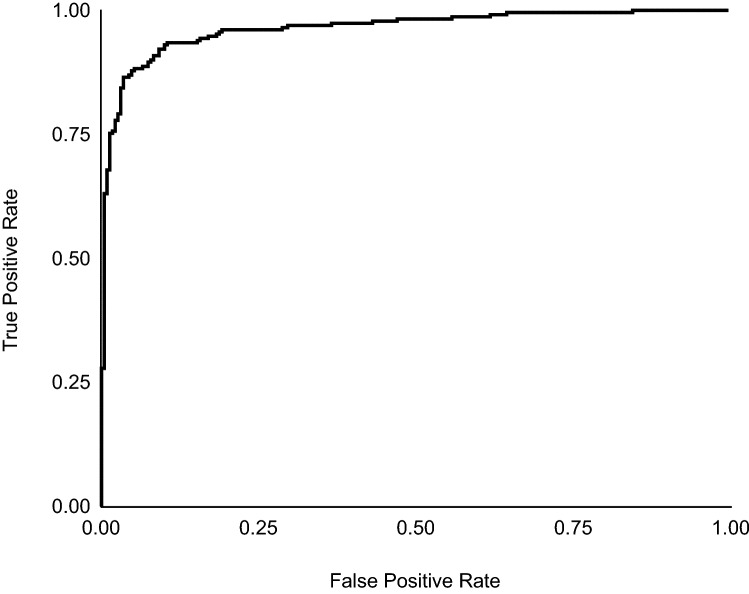
A receiver operating characteristic (ROC) curve showing the probability of the face encoder (*bearembed*) predicting matching (same individual) or non-matching pairs (different individual) of face chips under different thresholds. The steep curve towards the top left of the graph indicates high predictive utility at separating matching/non-matching pairs

For sub-application analysis using the test split of the Andean bear subset of the golden dataset (*n* = 121), the face encoder (*bearembed*) is effective at predicting matching and non-matching pairs with an accuracy of 90.9% (Table 4). A receiver operating characteristic curve (ROC) displays the performance of *bearembed* at different thresholds of true positive rate [TPR (recall/sensitivity)] and false positive rate [FPR (specificity); Fig. 5]. Further evaluation of *bearembed* using the two fivefold test regimes previously described, folds across all face chips and folds by ID label, shows mean accuracies of 90.3 ± 3.0% and 78.9 ± 5.5%, respectively (Table 4).

**Table 4:** Comparing the performance of the similarity comparison network across three test methods: golden test set, folds by face chips, and folds by ID label

Test method	Test n ± SD	Pairs *n*	Accuracy (%) ± SD	TPR (%) ± SD	TNR (%) ± SD	F1-score (%) ± SD
Golden test split	121	230	90.9	85.2	96.5	90.3
Folds by chip	121.8 ± 3.2	214	90.3 ± 3.0	83.4 ± 5.7	97.2 ± 0.9	89.5 ± 3.6
Folds by ID	121.8 ± 31	400	78.9 ± 6.3	75.2 ± 5.5	82.5 ± 9.5	78.1 ± 6.2

Pairs *n* = number of equal matching and non-matching pairs of face chips per test regime, *TPR* true positive rate, *TNR* true negative rate

The cumulative error of the face detection and embedding resulted in a drop in classification (*bearsvm*) accuracy from 89.3% (108 out of 121 correct IDs: golden dataset test) to 86.7% (104 out of 120 correct IDs).

## Discussion

Our multispecies facial detector network, trained on images of bears under human care, performed well, resulting in an average precision of 0.9–1.0 for every bear species used to train the network. Despite the relatively low number of training images (*n* = 1754 total) compared to large datasets typical of deep learning approaches, our results are comparable to single-species trained facial detection networks [African forest elephant *Loxodonta cyclotis*: 0.98, *n* = 1573 training images, Körschens et al. (2018); Western gorilla *Gorilla gorilla*: 0.91, *n* = 2000 training images, Brust et al. (2017); Giant panda: 1.0, *n* = 5854 training images, Chen et al. (2020)], and other detectors that focus on whole-body shape or focal body areas [Northern giraffe: 0.89 accuracy, Buehler et al. (2019); luderick *Girella tricuspidata*: 0.93, Ditria et al. (2020)]. Multispecies-trained detectors have more commonly been used in whole-body detection for species recognition, with performance in the range of 0.55 (AP_n_) to 0.97 (accuracy) (Loos et al. 2018; Norouzzadeh et al. 2018). MegaDetector (Beery et al. 2019b) is a multispecies (generalised) whole-body detector that can be used to remove empty frames and train project-specific classifiers from camera trap images. Our multispecies facial detector is intended to perform a similar function for the detection of faces for use in individual ID of bears, which could be replicated for other taxonomic families using our open source code (see Code Availability). Guo et al. (2020) recently developed a multispecies facial detector trained on 41 primate species and 4 carnivores resulting in detection accuracies of 0.91, 0.98, and 0.98 for golden snub-nosed monkeys *Rhinopithecus roxellana*, Tibetan macaques *Macaca thibetana*, and tigers *Panthera tigris*, respectively. In addition, Khan et al. (2020) present AnimalWeb, an annotated dataset of animal faces for 334 species across 21 orders, which achieves a class-wise face detection mean average precision of 0.64.

We only used images of bears where an individual identification was known, to avoid unintentionally training and testing a detector on images of a very low number of individuals, which could influence performance. This resulted in sample sizes being skewed by species, which could have biased the facial detector network in favour of those species with a larger dataset. However, two of the three species with the lowest sample sizes (American black bear, sloth bear), had the highest possible average precision (1.000), whereas the third species (Asiatic black bear) had the lowest average precision of all species (0.909), indicating the influence of additional variance beyond sample size. Further testing, on a larger sample size per species, should provide more information on the network's performance. In addition, due to the post hoc nature of image collection, we could not account for the date the images were taken across the whole dataset, leading to potential data leakage if images recorded on the same day were mixed between the training and testing datasets. Future research in this area should attempt to maintain a 24-h window (image independence) between image inclusion across the training or test datasets.

We have demonstrated how a detector trained across all eight bear species under human care, can be effective at detecting the faces of wild bears. This finding suggests that images of wildlife under human care may be useful in training deep-learning networks for use with images in field settings, whose collection can be challenging, and whose manual processing can be labor-intensive. We postulate that the relatively high average precision of our detector on wild brown bears could be due to variation in housing environments of the different species included in the dataset, as well as variation in facial biometrics from the inclusion of multiple species for training. Using a wild brown bear test dataset, when comparing the performance of a wild brown bear-trained detector to the performance of our new multispecies detector, precision was consistent between detectors (0.99 and 0.98, respectively), but recall was reduced (0.98 and 0.94, respectively). This suggests that the multispecies detector missed some faces of bears taken in situ, which may be due to additional background complexity (see Beery et al. 2018), pose variation, or size of the face within the image. The shape predictor improved at detecting the facial landmarks of bears under human care (all species combined: 0.083 ± 0.115; brown bears: 0.085 ± 0.103), compared to the shape predictor trained and tested on wild brown bears (0.111 ± 0.122: Clapham et al. 2020). However, when the multispecies shape predictor was tested on wild brown bears, its performance declined (0.161 ± 0.155), which could indicate a slight dissimilarity between the dataset of bears under human care compared to wild bears. Further analyses of performance should test images of wild bears for the other seven members of the *Ursidae*, to better evaluate its multispecies application in situ. Combining images of wildlife taken under human care with those taken in situ, or augmenting backgrounds of ex situ images (Beery et al. 2019a), could enhance the robustness of these datasets.

Using the multispecies face detector, we developed a pipeline for its use in automated individual identification of an example species, the Andean bear. The retrained face encoder (*bearembed*) obtained an accuracy of 90.9% for predicting matching and non-matching pairs of images of Andean bears in the golden test set. Five-fold analysis by chip and by label obtained accuracies of 90.3% and 78.9%, respectively, which outperforms the face encoder developed for wild brown bears for both methods (84.2% and 71.3%: Clapham et al. 2020). Other studies evaluating the performance of wildlife-focused deep learning networks for variants of face verification found similar or slightly reduced accuracies [lemurs *Lemuroidea* spp.: 83.1% (Deb et al. 2018); golden monkeys *Cercopithecus mitis kandti*: 78.7% (Deb et al. 2018); chimpanzees: 59.9% (Deb et al. 2018), 0.811 (mAP@1) (Schneider et al. 2020)], although our dataset has fewer images in comparison to these examples [this study: 609 images; Deb et al. (2018): 3000, 1450, and 5599 images, respectively; Schneider et al. (2020): 5599]. We suspect the facial markings of Andean bears may have contributed to the relatively high performance of our face encoder, but more test images are required to fully interpret its performance. Although our dataset is modest and we present this software as proof of concept rather than immediately applicable, the images used to train *bearembed* represent a long-term dataset of Andean bears under human care taken over at least 17 years. This undoubtedly added variation to the dataset beyond images taken on the same day, for example; especially as the facial appearance of Andean bears, and other bear species, can change over time (Yoshizaki et al. 2009; Van Horn et al. 2015; Clapham et al. 2020). The habitat of bear enclosures varied across the dataset, creating variation in the background of images, however, enclosure similarity within images of the same individuals may be a confounding variable positively influencing performance. While accuracy of *bearembed* was reduced when testing folds by ID label, it still shows good predictive utility (78.9%) for use in matching of new individuals. Face encoders that use similarity comparison networks, such as *bearembed*, are a promising tool for individual ID of wildlife due to their ability to generalise, allowing for the identification of new individuals that are not contained in the training dataset (Schneider et al. 2020). This is vital for wildlife studies looking to use automated approaches of individual ID for population assessments, such as spatial capture–recapture (SCR).

We developed a multispecies bear face detector using images of bears under human care that achieved a high level of performance across all eight bear species. This performance was transferable to a wild brown bear dataset, although further analysis using images of wild counterparts across all bear species is required to fully determine its application. Our automated end-to-end Andean bear example application correctly identified the individual in 85.9% of images inputted. These initial results indicate that a multispecies-trained face detection network can detect faces of a single species sufficiently to achieve high performance for individual classification. This could be important as within the bounds of human capacity, funding availability for wildlife research, and timeliness of conservation action required, it may not be possible to develop separate detectors and classifiers for every species at risk that could benefit from automated methods of individual identification. Automated methods are increasingly needed due to an ever-growing need for effective conservation (Dirzo et al. 2014) and expanding awareness that objective and replicable methods to identify individuals are needed to avoid undesired conservation outcomes (Choo et al. 2020; Johansson et al. 2020). Developing robust pipelines of automated individual ID will enable rapid and systematic data collection and processing on taxa of conservation concern, boosting the existing power of research to better inform conservation planning and management.

## Supplementary Information

Below is the link to the electronic supplementary material.

Supplementary file1 (PDF 195 kb)

## Data Availability

Not applicable.

## Code availability

*BearID* is an open‐source application available on GitHub at https://github.com/hypraptive/bearid (version 21.03). The pretrained models are available at https://github.com/hypraptive/bearid‐models (version 21.03).
