# Supplementary material for: Multispecies facial detection for individual identification of wildlife: a case study across ursids
Source: Mamm Biol. 2022 Apr 7;102(3):943–55. doi: 10.1007/s42991-021-00168-5 (PMC9499902; doi:10.1007/s42991-021-00168-5)
Supplement: Supplementary file 1 — Supplementary file1 (PDF 195 kb) [file 42991_2021_168_MOESM1_ESM.pdf]

Supplementary Material no. 1

Multispecies facial detection for individual identification of wildlife: a case study across ursids

Melanie Clapham*, Ed Miller, Mary Nguyen, Russell C. Van Horn

* melanie@bearid.org

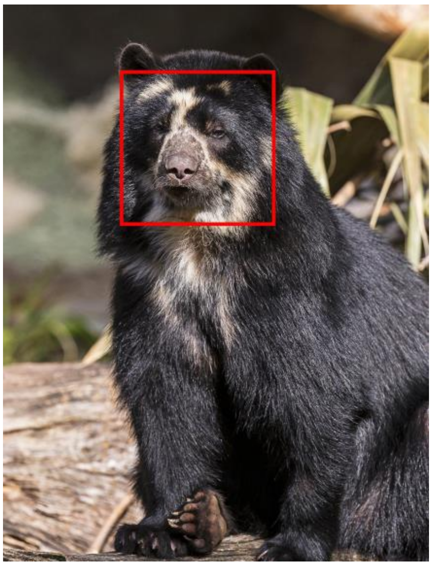

Highlights:

- Object detection is a critical component of individual-based photo-identification
- Software that functions across species could speed-up development and increase application
- We developed a face detector trained on images of all eight bear species taken under human care, and prototyped its use in individual ID of Andean bears
- The face detector maintained a high performance (>90%) across species and appears transferable to wild populations
- Multispecies face detection networks can perform sufficiently to allow for individual classification of a single species

This article is part of a thematic collection of articles (Special Issue) of *Mammalian Biology* and covers the following topics and taxa (marked with ☑) addressed in the Special Issue:

**Article Type**				
☐ Original Research	☑ Techniques	☐ Review	☐ Short Communication	☐ Concept Note

**Taxon**		**Topic**	
Terrestrial			
☐ Bats *(Order Chiroptera)*	☐ Primates : Great Apes *(Family Hominidae)*	☐ Acoustic ID	☑ Identification techniques
☑ Carnivores : Bears *(Family Ursidae)*	☐ Primates : Old World monkeys *(Family Cercopithecidae)*	☐ Aerial surveys	☐ Life-history
☐ Carnivores : Canids *(Family Canidae)*	☐ Ungulates : Bovids *(Family Bovidae)*	☐ Analytical innovations	☑ Machine learning
☐ Carnivores : Felids *(Family Felidae)*	☐ Ungulates : Deers *(Family Cervidae)*	☐ Automated pattern recognition	☐ Mark-recapture analysis
☐ Carnivores : Hyenas *(Family Hyaenidae)*	☐ Ungulates : Giraffes *(Family Giraffidae)*	☐ Behavioural ecology	☐ Morphometrics
☐ Carnivores : Mustelids *(Family Mustelidae)*	☐ Ungulates : Horses *(Family Equidae)*	☐ Camera-trapping	☐ Network analysis
☐ Elephants *(Family Elephantidae)*	☐ Multiple taxa *(3 or more Families/Orders)*	☐ Conservation management	☐ Photogrammetry
Marine		☐ Data management	☐ Population ecology
☐ Baleen whales : Right whales *(Family Balaenidae)*	☐ Large toothed whales *(Families Delphinidae & Hyperoodontidae)*	☐ Demographic parameters	☐ Site fidelity & Movement
☐ Baleen whales : Rorquals *(Family Balaenopteridae)*	☐ Pinnipeds : True seals *(Family Phocidae)*	☐ Field methodology	☐ Social ecology
☐ Carnivores : Bears *(Family Ursidae)*	☐ Porpoises *(Family Phocoenidae)*	☐ Genetic ID	☑ Software/Package development
☐ Carnivores : Mustelids *(Family Mustelidae)*	☐ Sirenians : Manatees *(Family Trichechidae)*	☐ Health conditions	☐ Thermal imagery
☐ Dolphins *(Family Delphinidae)*	☐ Multiple taxa *(3 or more Families/Orders)*	☐ Other: *(please specify)*	

References

Karczmarski L, Chan SCY, Rubenstein DI, Chui SYS, Cameron EZ (2022a). Individual identification and photographic techniques in mammalian ecological and behavioural research – Part 1: Methods and concepts. *Mammalian Biology* (Special Issue), 102 (3) https://link.springer.com/journal/42991/volumes-and-issues/102-3

Karczmarski L, Chan SCY, Chui SYS, Cameron EZ (2022b). Individual identification and photographic techniques in mammalian ecological and behavioural research – Part 2: Field studies and applications. *Mammalian Biology* (Special Issue), 102 (4) https://link.springer.com/journal/42991/volumes-and-issues/102-4
